# The changing causal foundations of cancer-related symptom clustering during the final month of palliative care: A longitudinal study

**DOI:** 10.1186/1471-2288-8-36

**Published:** 2008-06-04

**Authors:** Karin Olson, Leslie Hayduk, Marilyn Cree, Ying Cui, Hue Quan, John Hanson, Peter Lawlor, Florian Strasser

**Affiliations:** 1 Faculty of Nursing, University of Alberta, Edmonton, Alberta, Canada; 2 Sociology Department, University of Alberta, Edmonton, Alberta, Canada; 3 Faculty of Education, University of Alberta, Edmonton, Alberta, Canada; 4 Capital Health Regional Palliative Care Program, Edmonton, Alberta, Canada; 5 Cross Cancer Institute, Edmonton, Alberta, Canada; 6 Our Lady's Hospice, Harold's Cross, Dublin, Ireland; 7 Division of Palliative Care Medicine, Dept of Oncology, University of Alberta, Edmonton, Alberta, Canada; 8 Oncological Palliative Medicine, Section Oncology, Dept Internal Medicine, Cantonal Hospital, St. Gallen, Switzerland; 9 Palliative Care Center, Dept Internal Medicine, Cantonal Hospital, St. Gallen, Switzerland

## Abstract

**Background:**

Symptoms tend to occur in what have been called symptom clusters. Early symptom cluster research was imprecise regarding the causal foundations of the coordinations between specific symptoms, and was silent on whether the relationships between symptoms remained stable over time. This study develops a causal model of the relationships between symptoms in cancer palliative care patients as they approach death, and investigates the changing associations among the symptoms and between those symptoms and well-being.

**Methods:**

Complete symptom assessment scores were obtained for 82 individuals from an existing palliative care database. The data included assessments of pain, anxiety, nausea, shortness of breath, drowsiness, loss of appetite, tiredness, depression and well-being, all collected using the Edmonton Symptom Assessment System (ESAS). Relationships between the symptoms and well-being were investigated using a structural equation model.

**Results:**

The model fit acceptably and explained between 26% and 83% of the variation in appetite, tiredness, depression, and well-being. Drowsiness displayed consistent effects on appetite, tiredness and well-being. In contrast, anxiety's effect on well-being shifted importantly, with a direct effect and an indirect effect through tiredness at one month, being replaced by an effect working exclusively through depression at one week.

**Conclusion:**

Some of the causal forces explaining the variations in, and relationships among, palliative care patients' symptoms changed over the final month of life. This illustrates how investigating the causal foundations of symptom correlation or clustering can provide more detailed understandings that may contribute to improved control of patient comfort, quality of life, and quality of death.

## Background

### Symptom clustering

Symptoms seldom occur in isolation, and the coordination between symptoms has led to recent attempts to locate symptom clusters[1,2]. Developing strategies for assessing, investigating, and treating coordinated symptoms is an important clinical objective because it permits prioritization of symptom assessment and treatment. Responding to symptoms in isolation may lead to inappropriate treatment if the symptoms are interconnected by unknown underlying causal structures. Understanding the causes coordinating symptoms facilitates intervention by permitting the targeting of causally up-stream features so that any amelioration spreads to all the causally down-stream symptoms, thereby minimizing the number of required interventions.

Most symptom cluster research has focused on symptoms that occur in the context of active treatment [3-7] but a few studies have considered symptoms in individuals prior to treatment [7], or in those no longer receiving curative treatments [8,9]. Occasionally there are identifiable physiological foundations for symptom clusters [8] but more commonly, the co-occurrence of symptoms [4,6] and correlation between symptoms [5,9,10] is used to statistically create clusters. Factor analysis and principal component analysis [5,7] attempt to locate sets of highly inter-correlated symptoms. Unfortunately, researchers frequently fail to realize that a fundamental assumption of these analytic approaches is that there is a statistically-postulated common cause for each factor or component of clustered symptoms, and that no symptom within a cluster is permitted to causally influence any other symptom in that cluster. It seems unlikely, for example, that many readers recognized that Chow et al's [7] Component-1, which includes sense of well-being, pain, fatigue, and drowsiness, implicitly statistically forbids pain, fatigue and drowsiness from being causes of well-being, and statistically requires that these four symptoms became correlated primarily through their dependence on a single common cause.

Since one objective of studying symptom coordination is to locate effective treatments, it is critical that researchers do not inadvertently combine statistical analyses of symptoms in ways that are causally inconsistent. For example it is causally inconsistent to use factor analysis to claim that a certain set of symptoms are highly correlated and therefore form a cluster that should be treated as a unit, and then use other statistical methods such as ANOVA and regression to claim differences in the causes of symptoms within the cluster [5].

The appropriate research response seems clear – if we wish to investigate causal connections between symptoms, we need an analytic approach that does not statistically forbid the creation of such connections. This conclusion is consistent with the call by Barsevick et al [9] for use of path and structural equation models in symptom cluster research. Both these styles of analysis seek causal structuring and control for potentially confounded variables, but structural equation modeling is superior because it permits adjustment for measurement error, provides a test of the model, and produces diagnostics relevant to model reassessment. Structural equation modeling is superior to other analytic approaches such as regression and ANOVA because it prods the researcher to theorize holistically about the full set of modeled symptoms.

The initial model that was proposed for this study was drawn from our clinical observations of patients on an acute palliative care unit. We thought we could see evidence of connections between symptoms but noticed that the nature of the symptom connections seemed to change during the last month of life. We decided to use structural equation models to gain a more detailed understanding of the causal foundations of the symptoms reported by patients. We thought that changes in causal relationships among the symptoms would underlie the changes in the connections between symptoms.

### Conceptual model

Normally the effects specified in structural equation models are drawn from published reports of other studies. In our case, the dearth of relevant causally-oriented research necessitated that relationships in our model be based on our clinical observations and experience. The symptoms included in our model were those that commonly occur in palliative patients, as measured using the modified Edmonton Symptom Assessment System (ESAS), an instrument comprised of eight symptoms and well-being, all measured using numerical rating scales [11].

**Figure 1:**
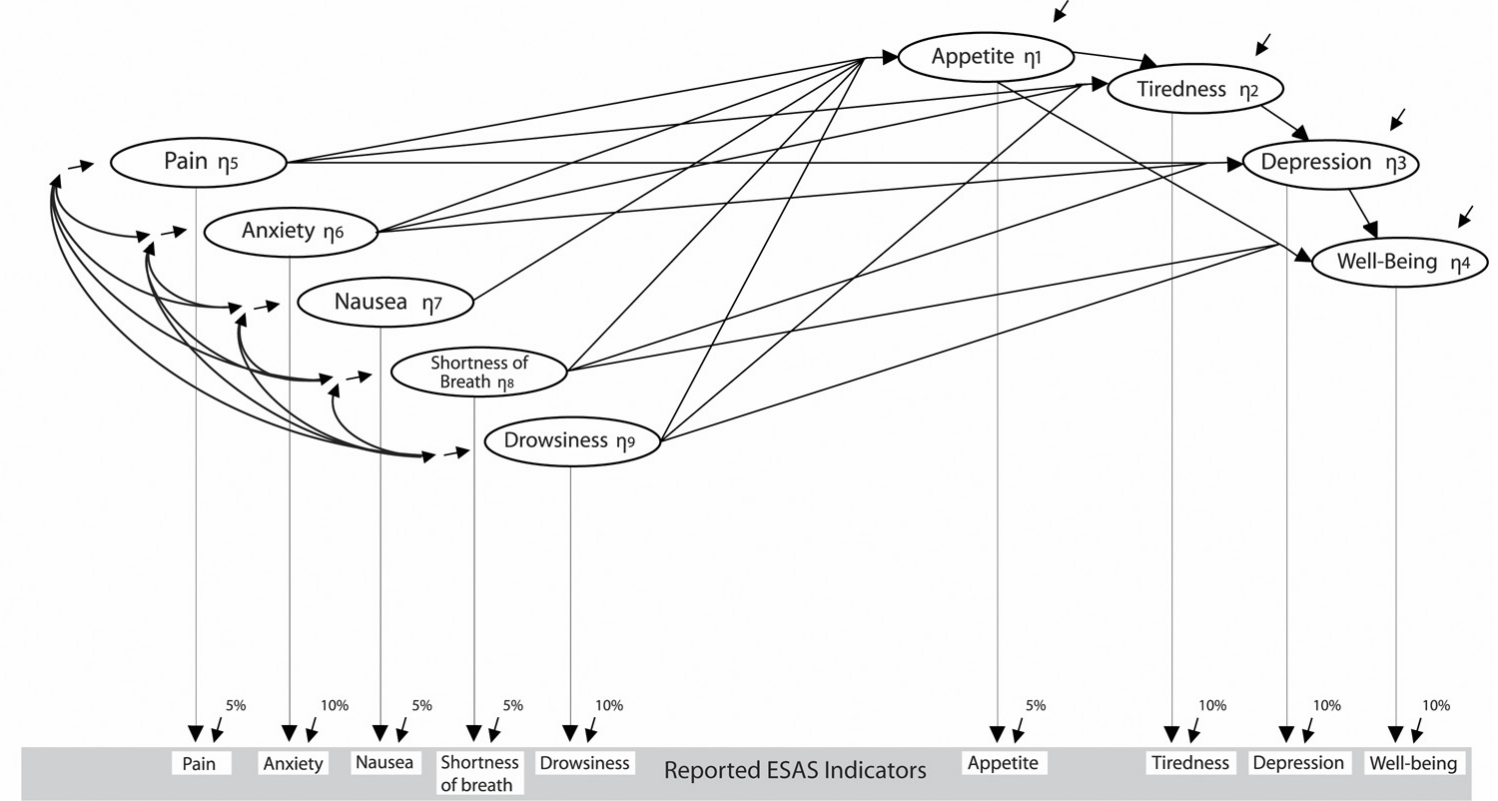
The basic symptom model.

Based on clinical experience and symptom pathophysiology, we classified the 9 items from the ESAS into two categories (see Figure 1). Pain, anxiety, nausea, shortness of breath and drowsiness were selected as exogenous or background variables. Appetite, tiredness (fatigue), depression, and well-being were specified as endogenous or dependent variables. Although the measure of depression in the ESAS is more akin to a downturn in mood as opposed to a clinical state as outlined in Diagnostic and Statistical Manual of Mental Disorders (DSM) IV [12], the decision to include depression as an endogenous variable was based on Post's kindling theory [13], the work of Francoeur [8] with advanced cancer patients, and the Edmonton Fatigue Framework recently published by our group [14].

The variances of, and covariances between, the endogenous variables were modeled as arising from effects originating in the exogenous variables, and/or effects of other endogenous variables. The error term attached to each endogenous variable encapsulates the net impact of all unidentified causal factors. The model does not examine the causal sources of the coordination between the exogenous variables but it examines the effects of the exogenous variables, and it both permits and statistically adjusts for covariances between these variables. The locations of the potential effects specified in the model were driven by our best clinical assessments but we nonetheless anticipated that the data might prompt inclusion of a few additional effects, or speak against some included-effects by providing insignificant effect estimates.

## Methods

### Study sample

Following receipt of ethical clearance from the Health Research Ethics Board at the University of Alberta, unidentified patients who met eligibility criteria were selected from the symptom control database of the Capital Health Regional Palliative Care Program (CHRPCP). All patients were admitted to either the inpatient palliative unit or a hospice within the CHRPCP from 1995–2000, and had complete Edmonton Symptom Assessment System (ESAS) scores on both day seven of the fifth week before death (1 month) and day seven of the second week (one week) before death. In addition, all patients had a Mini Mental Status Exam (MMSE) score of at least 22/30 within three days of both data collection points, since the MMSE was routinely completed twice per week at that time. A Mini Mental Status Exam score of at least 22/30 is routinely used in our setting to indicate that patients' cognitive abilities enable them to provide valid responses as they personally complete the ESAS. All patients were receiving symptom control interventions and other supportive and palliative care treatments, but were not receiving any anticancer treatments. All data (diagnoses, age, gender, symptom scores, and well-being scores) were obtained from the patient database maintained by the CHRPCP.

### Measures

Patients reported their current (at that moment) pain, tiredness, nausea, depression, anxiety, drowsiness, appetite, shortness of breath and well-being using either the earlier (1992–1999) 100 millimetre visual analogue, or the more recent (since 1999) numerical rating scale version of the ESAS. Several research groups have shown that the ESAS is both reliable and valid [15-17]. The low end of both versions of the ESAS was anchored by the word "absent" or, in the case of well-being, "best possible", while the high end of the symptom and well-being scales were anchored by "worst possible." Patients using the visual analogue ESAS placed a mark on a 100 millimeter line corresponding to the intensity of each of the eight symptoms and their well-being. The distance from 0 to the mark was measured in millimeters and rounded to the nearest 10 (such as 10, 20, 30). In the numerical rating version patients circle a number from 0 to 10 (such as 1, 2, 3) that best described the intensity of each symptom and well-being. The numerical rating scale scores were multiplied by 10 prior to entry into the CHRPCP database to provide them a 0 to 100 range comparable to that of the visual analogue scale. At the time the study data were collected, the ESAS was completed once per day in the hospices and twice per day in the inpatient palliative unit. If two ratings were provided, the average of these two scores was used.

### Analysis

Covariance matrices were constructed for the measures obtained on day seven of week five (one month) and day seven of week two (one week) before death, and were used as input for Version 8.72 s of the Linear Structural Relations (LISREL) program [18] to obtain maximum likelihood estimates of the coefficients in the Figure 1 model. Measurement error variances were established based on the expected accuracies of measurement. The measurement error variance was set at 5% for symptoms we thought were easier for patients to assess, such as appetite, pain, nausea, and shortness of breath. The measurement error variance was set at 10% for symptoms we thought were more difficult for patients to assess, such as tiredness, drowsiness, depression, anxiety, and well-being. The model revisions outlined below were made based on large modification indices whenever the coefficients were theoretically plausible and the model remained stable. Readers unfamiliar with structural equation modeling might consult Hayduk [19] or Bollen [20] and those desiring background on measurement error adjustments in the context of single indicators might see Hayduk [19,21].

## Results

Of the 140 patients who had MMSE scores of 22/30 or higher, complete sets of scores (all 8 symptoms plus well-being) were available for 82 individuals. These individuals ranged in age from 37 to 93 years with a mean age of 64. Forty-nine (60%) subjects were females. The majority of patients were diagnosed with one of four cancers: 27% with gastro-intestinal cancer; 21% with genito-urinary cancer; 20% with lung cancer; and 12% with breast cancer. Means and standard deviations for the symptom scores at both one month and one week before death are shown in Table 1.

**Table 1:** Means and standard deviations of symptom scores one month and one week before death

Symptom	One Month	One Week
	Mean (s.d.)	Mean (s.d.)
Appetite	46.8 (29.9)	60.3 (32.1)
Tiredness	43.2 (20.0)	58.5 (25.0)
Depression	32.9 (22.7)	38.5 (29.1)
Well-being	42.4 (23.5)	49.0 (23.4)
Pain	37.5 (22.7)	44.0 (26.9)
Anxiety	33.4 (24.0)	38.1 (28.5)
Nausea	18.4 (16.0)	25.7 (24.9)
Shortness of Breath	24.0 (21.1)	31.7 (27.2)
Drowsiness	39.8 (22.5)	55.6 (27.7)

Standard deviations (s.d.) in parentheses; symptoms scored 0 = best possible to. 100 = worst possible

Notice that there is substantial variability in all the measurements at both time points, and that all symptoms tended to became more intense and more variable as the time of death approached.

The covariance matrices used in estimating the models are provided in Table 2. Table 3 contains the estimates for the coefficients in the two models, and it is to these that we now turn.

**Table 2:** Indicator Covariance Matrices and Latent Exogenous Variable Correlations

Symptom	Appetite	Tired-ness	Depression	Well-being	Pain	Anxiety	Nausea	Short of Breath	Drowsiness
	One Month Before Death (n = 82)								

Appetite	895.3								
Tiredness	242.4	399.6							
Depress.	296.1	188.5	513.6						
Well-being	427.5	218.8	320.6	550.1					
Pain	370.8	221.8	231.3	255.7	514.4	**0.47**	**0.41**	**0.40**	**0.48**
Anxiety	336.0	110.6	388.1	390.7	236.9	577.2	**0.50**	**0.14**	**0.57**
Nausea	172.4	97.6	177.7	144.6	153.3	190.3	297.2	**0.16**	**0.40**
Short of Breath	8.5	87.5	43.0	70.3	181.2	66.3	54.6	443.5	**0.19**
Drowsy	353.4	332.4	301.2	321.4	227.3	275.9	143.0	85.0	505.8

	One Week Before Death (n = 82)								

Appetite	1029.2								
Tiredness	318.4	623.3							
Depress.	250.5	261.9	848.6						
Well-being	426.3	262.9	446.2	549.8					
Pain	166.4	310.1	276.5	226.1	715.9	**0.40**	**0.37**	**0.42**	**0.43**
Anxiety	205.2	241.8	661.9	372.4	280.1	811.5	**0.65**	**0.33**	**0.49**
Nausea	150.7	177.6	423.6	300.1	234.4	423.5	620.8	**0.39**	**0.39**
Short of Breath	204.4	164.4	347.7	227.4	288.5	243.8	252.2	739.6	**0.32**
Drowsy	406.6	522.2	359.0	376.6	295.1	345.6	250.5	226.7	765.1

*Indicator variances on the diagonal, indicator covariances below the diagonal, and correlations of the corresponding latent exogenous variables obtained from LISREL's standardized phi matrix in bold above the diagonal. Correlations between any pair of latent indicators can be calculated from the lower triangular entries by dividing the covariance between the indicators by the square root of the product of the two relevant variances $\text{Cov}(\text{XY})/\sqrt{\text{Var(X) Var(Y)}}$

**Table 3:** Unstandardized Direct Effects in the Structural Equation Models

To	Time Until Death	From									R^2^
		
		Appetite	Tired	Depress	Well-Being	Pain	Anxiety	Nausea	Shortness of Breath	Drowsy	
Appetite	1 Month	--	--	--	--	0.60*	0.18	0.01	-0.35*	0.43*	0.51
	1 Week	--	--	--	--	-0.06	0.01	-0.03	0.14	0.58*	0.26
Tired	1 Month	-0.05	--	--	--	0.25*	-0.31*	--	--	0.84*	0.80
	1 Week	0.02	--	--	--	0.17*	-0.08	--	--	0.71*	0.73
Depress	1 Month	--	0.32*	--	--	0.03	0.68*	--	-0.09	--	0.71
	1 Week	--	0.07	--	--	-0.06	0.84*	--	0.22*	--	0.83
Well-Being	1 Month	0.21*	--	-0.01	--	--	0.46*	--	0.04	0.28*	0.72
	1 Week	0.26*	--	0.42*	--	--	--	--	-0.01	0.17*	0.72

*Coefficient is more than 2.0 standard errors from zero (significant at the 0.05 level)

-- Denotes coefficients that were excluded (zero) by model specification

### One month (day 7 of week 5) before death

Our initial modeling attempted to be conservative by permitting all the endogenous variables' error terms to covary but this resulted in large yet insignificant covariances suggestive of colinearity. We removed the correlations among the error terms and respecified the model as an all-η model [19] in order to gain more detailed diagnostic information. The resulting modification indices suggested an additional direct effect of appetite on well-being. Since this was clinically reasonable, this effect was included, as shown in Figure 1, and resulted in a reasonable fit between the one-month data and the model's implications (χ^2 ^= 14.2 with 9 degrees of freedom; p = 0.11).

**Figure 2:**
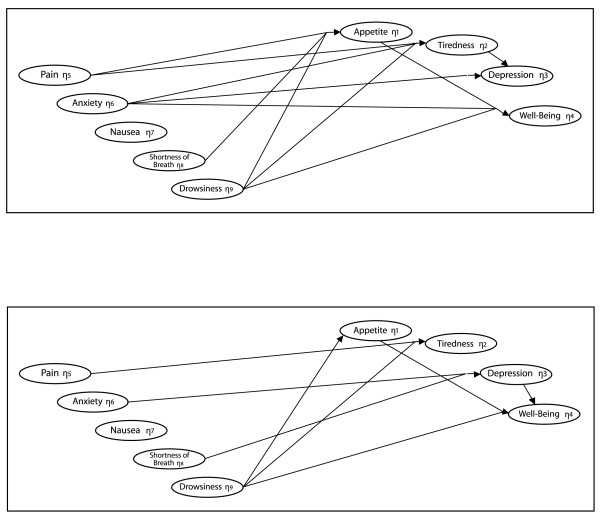
Significant* effect estimates. Top figure: One month before death. Bottom figure: One week before death. Note: *An effect coefficient exceeding two standard errors is reported as statistically significant.

Although the fit between the one-month data and the Figure 1 model was now "acceptable" in the sense that χ^2 ^did not detect significant inconsistencies, this model underwent one additional revision. Based on the modification indices, a theoretically reasonable effect of anxiety on well-being was added and resulted in χ^2 ^= 4.9 with 8 degrees of freedom, p = 0.77. No further changes were made to this model. The estimates of the effects in this model are included in Table 3, and the significant effects are presented in the top of Figure 2.

This model explains over 70% of the variation in each of two symptoms (tiredness and depression) as well as well-being (Table 3). The strongest effects in this model were those connecting anxiety to depression (0.68, standardized 0.72) and drowsiness to tiredness (0.84, standardized 0.94). The two significant predictors of depression (anxiety and tiredness) are the major contributors to the 71% explained variation in depression. Eighty percent of the variation in tiredness was primarily attributable to drowsiness, pain, and anxiety. Well-being improved when appetite improved and when there was decreased anxiety and drowsiness, which collectively account for about 72% of the variance in well-being. Fifty-one percent of the variation in appetite was explained, primarily by the significant effects from pain, drowsiness, and shortness of breath.

### One week (day 7 of week 2) before death

Despite what would traditionally be viewed as excellent fit between the data and the original one-week model (χ^2 ^= 5.3 with 9 degrees of freedom; p = 0.80), we reviewed the diagnostics for this model. The largest modification index suggested an effect from well-being to anxiety, which might be clinically possible, but the estimate of this effect was insignificant and statistically unstable, so no revisions were made to the Figure 1 model for one week before death.

In agreement with the one-month model, the positive effects leading from anxiety to depression and from drowsiness to tiredness remained very strong (Table 3). Because a one unit increase in anxiety causes nearly a corresponding unit increase in depression (0.84, standardized 0.82), anxiety contributes most of the explained (83%) variation in depression. Drowsiness (primarily) and pain (slightly) explained nearly three quarters (73%) of the variation in tiredness. A combination of appetite, depression, and drowsiness accounted for 72% of the variation in well-being. Drowsiness was the only variable contributing significantly to the 26% explained variation in appetite.

### Considering the groups together

Of the 17 effects postulated in the baseline model (Figure 1), 12 (or 71%) produced significant effect estimates in one model or the other – hence the baseline model was comfortably but not excessively parameterized. And, as we had anticipated, there were some substantial shifts in the patterning of effects during the last month of life. If the effects common to the models are constrained to be equal (in a multi-group model) this results in significant inconsistencies between these models and the data (the difference-χ^2 ^= 35.9, df = 17, p < 0.01), so there is clear evidence that at least some of the effects have changed between one month and one week prior to death.

Only three effects remained strong and stable throughout the two time periods included in this study. Drowsiness consistently led to both decreased appetite and increased tiredness, and anxiety consistently contributed to depression (Table 3). From these data we cannot tell what the participants were anxious about (impending death, family, financial or estate matters) but it is clear that anxiety was by far the strongest source of depression throughout the time frame covered by this study.

The one-month model displayed the most frequent, and the most wide-spread, effects (Figure 2). The one-week model contained fewer significant effects, and the pattern of effects changed importantly during the period from one month to one week before death. Pain, for example, had a significant effect on both appetite and tiredness at one month before death, but only had a significant effect on tiredness by one week before death. The medical control of pain in these facilities is state of the art, but improvements in pain control over the final weeks of life do not explain the declining effects of pain. Table 2 indicates that the overall level of pain tended to increase and become more variable over this period – so pain's lack of effects is not due to either the absence of pain or to restrictions on the range of pain scores.

We were surprised to observe that nausea had no effect on appetite at either time period, despite considerable variability in the severity of nausea scores both within and between time periods (Tables 1 and 3).

Some of the most intriguing shifts that occurred over the last month of life concerned depression, well-being, and anxiety. Notice the absence of an effect of depression on well-being in the one-month model, and the presence of a significant effect of depression on well-being in the one-week model. At one week, anxiety still has a strong effect on depression but its direct effect on well-being is gone. (The modification indices did not call for this effect and estimating this effect resulted in an estimate that was far from significant, and did not importantly change any other estimates.) Thus there is a subtle but important shift from anxiety causing both depression and well-being at one month before death, to anxiety having an effect on well-being through depression at one week before death. The correlation between depression and well-being can be calculated from Table 2 as 0.60, 0.65 (for the one-month and one-week models respectively), but our model shows that only the correlation at one week is consistent with an effect of depression on well-being. A clinician who noticed the stability in the correlation between depression and well-being could not be expected to intuit that only the correlation at one week before death provided significant evidence of an effect of depression on well-being. Here we see a relatively stable correlation between depression and well-being emerging from a changing causal foundation. Another way to consider this is to focus on the effect of anxiety on well-being. At one month before death the model diagnostics prodded the inclusion of a direct effect of magnitude 0.46 with no indication of any indirect effect through depression because the effect of depression on well-being is nearly non-existent. At one week the appearance of a substantial effect of depression on well-being (0.42) results in there being a substantial indirect effect of anxiety on well-being via depression (0.84 × 0.42 = 0.35) that is nearly as strong as the direct effect of anxiety on well-being in the one-month model. At one month before death, depression is not importantly implicated in anxiety's effect, but at one week before death depression has transformed into being a major carrier of anxiety's effect on well-being. These findings suggest that addressing depression could effectively control the effects of anxiety on well-being at one week but not at one month.

Most of the negative effects in Table 3 are of little interest since these are likely to be mere sampling fluctuations around true null effects, but two significant negative effects deserve comment. The first of these, the negative effect of shortness of breath on appetite at one month, fits with our clinical observations that shortness of breath is exceptionally distressing, even when intensity scores are relatively low and that many individuals associate the ability to eat with the ability to fight the disease process. We suspect that the interference of low intensity shortness of breath on eating may trigger a fear of not eating enough and thus a desire to eat more, independent of the usual mechanisms that control appetite. By one week before death, despite a continued decline in both shortness of breath and appetite, we found a shift to a non-significant positive relationship. We attribute this change to the growing effect of drowsiness on appetite, and suspect that at this point in the illness trajectory, patients were simply too drowsy to want to eat, irrespective of any distress associated with shortness of breath (Table 1).

The other significant negative effect is that of anxiety on tiredness at one month. We suspect that this relationship is medication-based because medications that reduce anxiety tend to increase tiredness, but this would not explain why the effect would weaken by one week despite increases in the levels of both anxiety and tiredness (Table 2). It is possible that the differential size of the negative effect estimates at one month and one week are sampling fluctuations around a true negative effect that lies between the two negative estimates, so we are inclined not to emphasize this particular shift in effects.

Notice the modest and contrasting indirect effects that anxiety and pain have on depression through tiredness. At one month before death, the negative effect means anxiety works through tiredness to slightly decrease (or relieve) depression (-0.31 × 0.32 = -0.10), while pain's positive effect works through tiredness to slightly increase depression (0.25 × 0.32 = 0.08). Tiredness seems to simultaneously act as a releaser of anxiety's, and as a propagator of pain's, impact on depression at one month before death. Both these effects are disrupted at one week before death by the erosion of the effect of tiredness on depression.

There has been considerable debate among clinicians about whether depression is a cause of tiredness in this population. Our model postulates and provides evidence consistent with the opposite of this, namely that tiredness causes depression. The effect from depression to tiredness was well identified in our models but the modification indices did not suggest insertion of this effect and the effect was not significant if inserted in either the one week or one month model. This suggests that while the management of depression is an important clinical objective, it should not be expected to decrease tiredness.

### Correlations among the exogenous latent variables

The background correlations among the exogenous latent variables (Table 2 above the diagonal and depicted in Figure 1 but omitted from Figure 2 for legibility) contribute to the connections between the endogenous symptom variables because correlations among the causes lead to correlations among their effects. These exogenous variable correlations are substantial enough to contribute materially to connections among the endogenous symptoms. The minor, but possibly non-negligible, changes in these exogenous correlations between the one-month and one-week models may shroud additional causal changes that contribute to changes in the patterns of correlations among the endogenous symptoms in our model. Our models acknowledge and statistically adjust for the exogenous correlations, and changes in correlations, but they do not attempt to delineate the specific causal connections producing the exogenous variables' correlations. Hence, the variations in the exogenous variables' correlations, though minor, are possibly pointing to additional currently unexamined causal changes contributing to changes in symptom correlations.

Notice that even a substantial correlation, or change in correlation, between a pair of exogenous variables neither demands nor forbids consistency in the effects these variables have on the endogenous symptoms in the models. Locating specific exogenous causal changes would add to, or supplement, rather than demand revision of, the causal features that are producing the connections between the endogenous symptoms in our models.

## Discussion

The search for causal forces that provide the coordination between symptoms is an alternative to the current approach to the study of symptom clusters. We consider the correlations between symptoms to be artifacts that are created through the action of underlying causal effects. From this perspective, if the relevant causal forces change, the symptoms display new patterns of correlations. Studies using factor analysis presume that a stable common-cause underlies the symptoms in any given factor. We, on the other hand, remain receptive to the possibility that different and even changing causal structures underlie the connections between symptoms. By continuing to examining the causal networks underpinning a variety of symptoms, we hope to eventually target interventions that effectively address coordinated sets of symptoms. Symptoms reflecting a common cause would be most effectively addressed by proper management of that common cause, and symptoms linked in a causal chain would be most effectively addressed by proper management of the symptom heading the causal chain.

Early studies on symptom clusters [1,2] were silent regarding the stability of symptom coordinations, but recently Chow and colleagues [7] used factor analysis to show that symptoms loading on various factors changed over time. They found that sometimes anxiety and depression loaded by themselves, and at other times anxiety and depression loaded with well-being, fatigue, or drowsiness, but they did not model the causal foundations of these shifts. We found that the effects coordinating some symptoms were stable over time, while others were not. Specifically, we found that the effects of anxiety on depression, of drowsiness on tiredness, and of appetite on well-being were stable, while the effects of pain on appetite, anxiety on well-being, and depression on well-being were not stable. Based on these findings, some may consider that we identified three clusters: anxiety/depression, drowsiness/tiredness, and appetite/well-being. These three pairs of symptoms do not constitute symptom clusters in our view. Rather, they are simply pairs of symptoms in which changes in the first symptom consistently lead to changes in the second symptom at two points in time. We do not know whether these relationships would hold over a longer period of time.

This study is the first to provide quantitative evidence of a stable causal relationship between appetite and well-being, but this effect is not surprising. A number of research groups have reported distress associated with loss of appetite [22-25]. In addition to the physiological benefits of the intake of nutrients, clinicians have noted the social importance of appetite. Appetite makes it possible for patients to share meal times with family and friends in a manner that feels "normal".

A number of authors have shown that anxiety and depression are common among palliative patients [26-28]. Wilson and colleagues reported that 24% of the participants in their sample of palliative care patients met the criteria for at least one anxiety or depressive disorder [29]. The causes of anxiety and depression in this population range from medical complications to psychological and existential concerns [30], and anxiety and depression are often accompanied by the somatic complaints associated with advanced disease. These complexities make it tempting to fall back on common sense by viewing anxiety and depression as merely "somehow similar." A preferable approach is to tame the complexity by incorporating it into an appropriately structured model. Our clinical observation of patients moving into depression pointed to anxiety as a source of depression, irrespective of the multiplicity and diversity of additional sources of both depression and anxiety. Our model's specification mirrors what we saw at the bedside, regardless of the additional and even unknown factors that influence both anxiety and depression among palliative patients, anxiety led to depression. An insignificant effect estimate could have spoken against our understanding, but the estimate for the effect of anxiety on depression was significant at both one month and one week before death, suggesting effective management of anxiety would help reduce depression. Depression's subsequent but changing effect on well-being suggests that the degree to which anxiety management will carry over to patients' perceptions of their well-being depends on the proximity to death, but given the potential for at least some improvement in well-being, this seems worthy of future investigation.

Our modeling of drowsiness and tiredness provides an instance where we can informatively follow a reviewer's suggestion to clearly differentiate between common sense and our model's specification. We modeled drowsiness as a cause of tiredness. From a common sense perspective, one may think of drowsiness and tiredness as synonyms for sleepiness, and hence interpret our model as saying sleepiness causes sleepiness. Drowsiness as measured by the ESAS was conceptualized as medication or disease-induced neurological interference which could occur throughout the day and not only at the transition from wake to sleeping, Tiredness as measured by the ESAS was conceptualized as the lack ability to engage in desired activities, even when fully awake. Patients are regularly reminded of these important distinctions when we ask them to complete the ESAS. Thus the effect of drowsiness on tiredness is more akin to an effect of mind on body, than to the common-sense understanding of drowsiness and tiredness as sleepiness.

### Limitations and Strengths

This study is limited in that we had too few cases to be able to differentiate among types of cancer. Since type of cancer may influence symptom profile (e.g., breathlessness may be more common in lung cancer than in prostate cancer), there remains the possibility of finding even tighter symptom interconnections and simpler causal models for specific cancer types. The patient's type of cancer is not likely to change importantly in the last weeks of life, so even if we had included cancer diagnoses in our analysis, it would not have accounted for the changing patterns of effects observed in our models. In the future, we hope to investigate models of the symptoms displayed by different types of cancers via "stacked" or multi-group structural equation models.

The majority of our data were collected on an acute palliative unit or in hospice units in extended care centers. Thus the extent to which these findings generalize to other palliative care settings is unknown but could be addressed in future studies by collecting comparable data in home care and other hospice environments.

The two data collection points in this study were fairly close together. In a future study we plan to examine additional data collection points spanning a longer time period. Also, complete data were only available for only 82 of 140 potential participants and the symptoms that were assessed were limited to those measured by the ESAS. It is possible that results might differ with use of other assessment tools, or more cases. Each of the items included in the ESAS is far more complex and multifaceted than can be represented fully by a numerical rating scale. For example, recent developments in the assessment of advanced cancer pain suggest that in order to accurately assess pain, one must consider the mechanism of pain, the degree to which pain is a function of movement, related psychological distress, history of alcohol or drug addiction, and cognitive function [31,32]. The theoretical needs of research with palliative patients, however, must be weighed against patient burden. The ideal data collection strategy for pain and other symptoms may require more effort than palliative patients can provide.

It is possible, but unlikely, that measurement of symptoms beyond those available in the ESAS would have altered the main findings of this study. New measures of variables that are causally down-stream from the variables in our model would by definition be incapable of influencing the variables in our model. New measures of causally up-stream variables that influence just the exogenous variables would have only more clearly specified the sources of the currently-free covariances between these variables. New variables influencing only specific endogenous variables would have replaced some of the modeled error variables and hence increased the proportion of explained variance in these variables but the current effect estimates would not change. Our primary concern was about whether symptoms that were common causes of two endogenous symptoms or one exogenous and one or more endogenous symptoms were missing because this would have rendered the model misspecified. While this is the most challenging kind of concern, the fact that both models fit with minimal model revision constitutes evidence that no such causal features are required. If such a symptom had been missed, the models would have failed to fit and would have provided covariance residuals diagnostically indicative of the location of the missed variable's effects but this did not happen, so this possibility seems unlikely in light of the available evidence.

Our above comments alluded to the detailed attention required in setting up an original structural equation model. This can be seen as a limitation of the method because it requires substantially greater effort and thoughtful engagement than is required by more exploratory methods. But encouraging thoughtful engagement with the relevant substantive variables can also be viewed as a great strength. The model examined in this study was constructed by several members of the study team over a period of several months, based on their clinical observation that as patients approached death, there were changing patterns in the patients' ESAS symptom profiles. We wondered about whether the patterns were real and if so, how the symptoms included in the ESAS were related to each other and to well-being. Structural equation modeling provided an opportunity to test ideas that came from our observations and discussions. From a treatment perspective, considerable advantage is gained by encouraging researchers to propose and test specific thoroughly-considered causal structures rather than stopping with the identification of correlated symptoms.

The decisions regarding which variables to model as exogenous or endogenous, and which specific effects to included or excluded, were not difficult in our case but we might have felt differently had our model failed. A strength of structural equation modeling is that it provides an opportunity to test ideas. The model may either fit the data, as it did in our case or not fit the data and be considered a "failed" model. Some researchers may be reluctant to expose their ideas to this kind of risk. The model test and diagnostics cannot detect all model specification problems [19] but the testing is strong enough that passed model tests (as in our models) provide some substantial reassurance unavailable with procedures like factor analysis and regression.

An additional methodological strength of our study design was that the data focus on fixed periods before death. This approach assisted in the identification of causal structures because the participants at each time point were more homogenous in their disease process than are typical palliative populations or participants recruited at the time of diagnosis or treatment.

## Conclusion

Our findings suggest that some of the causes that coordinate symptoms in palliative care patients remain stable between one month and one week prior to death (specifically the impact of anxiety on depression, drowsiness on tiredness, and appetite on well-being), while other causes (specifically the effect of pain on appetite, and of anxiety on well-being; and the emergence of an effect of depression on well-being) do not remain stable.

At any point in the palliative treatment trajectory, an intervention that reduces anxiety is likely to decrease depression, but how this improves the patients well-being depends on the proximity of death. Our models suggest that one month before death an intervention reducing anxiety would directly contribute to improving well-being, but at one week a reduction in anxiety would work through reduced depression to produce well-being.

Tailoring interventions to capitalize on operative causal forces should optimize effectiveness, but we hesitate to provide specific advice on the basis of a single study. Instead, we urge further attention to the causal foundations that give rise to the connections between symptoms in specific disease groups. Identifying group-appropriate and time-point-appropriate causal foundations of the connections between symptoms should maximize clinical effectiveness and improve patient comfort, quality of life, and quality of death.

## Authors' contributions

KO conceived and designed the study, arranged for the data acquisition, contributed to the interpretation of the results, drafted and critically revised the manuscript, and gave final approval of the manuscript for publication. LH contributed to the data analysis and interpretation, drafting and critically revising the manuscript, and gave final approval of the manuscript for publication. MC contributed to the interpretation of the results, drafted and critically revised the manuscript, and gave final approval of the manuscript for publication. YC contributed to the data analysis and gave final approval of the manuscript for publication. HQ provided the data and gave final approval of the manuscript for publication. JH assisted with analysis and interpretation of the data and gave final approval of the manuscript for publication. PL assisted in developing the causal model and gave final approval of the manuscript for publication. FS critically revised and provided comments on the manuscript and gave final approval of the manuscript for publication.

## Declaration of competing interests

The authors declare that they have no competing interests.

## Pre-publication history

The pre-publication history for this paper can be accessed here:


